# Outcomes in Patients with *FLT3-*Mutated Relapsed/ Refractory Acute Myelogenous Leukemia Who Underwent Transplantation in the Phase 3 ADMIRAL Trial of Gilteritinib versus Salvage Chemotherapy

**DOI:** 10.1016/j.jtct.2022.12.006

**Published:** 2022-12-13

**Authors:** Alexander E. Perl, Richard A. Larson, Nikolai A. Podoltsev, Stephen Strickland, Eunice S. Wang, Ehab Atallah, Gary J. Schiller, Giovanni Martinelli, Andreas Neubauer, Jorge Sierra, Pau Montesinos, Christian Recher, Sung-Soo Yoon, Yoshinobu Maeda, Naoko Hosono, Masahiro Onozawa, Takayasu Kato, Hee-Je Kim, Nahla Hasabou, Rishita Nuthethi, Ramon Tiu, Mark J. Levis

**Affiliations:** 1 Abramson Cancer Center, University of Pennsylvania, Philadelphia, Pennsylvania; 2 Division of the Biological Sciences, University of Chicago, Chicago, Illinois; 3 Department of Internal Medicine, Yale School of Medicine, New Haven, Connecticut; 4 Department of Internal Medicine, Vanderbilt-Ingram Cancer Center, Nashville, Tennessee; 5 Department of Medicine, Roswell Park Comprehensive Cancer Center, Buffalo, New York; 6 Division of Hematology and Oncology, Medical College of Wisconsin, Milwaukee, Wisconsin; 7 Division of Hematology and Oncology, David Geffen School of Medicine at UCLA, Los Angeles, California; 8 IRCCS Istituto Scientifico Romagnolo per lo Studio dei Tumori “Dino Amadori” IRST S.r.l, Meldola, Italy; 9 Universitätsklinikum Giessen und Marburg GmbH, Marburg, Germany; 10 Hospital de la Santa Creu i Sant Pau and Josep Carreras Leukemia Research Institute, Barcelona, Spain; 11 Department of Hematology, University Hospital La Fe, Valencia, Spain; 12 Centre Hospitalier Universitaire de Toulouse, Institut Universitaire du Cancer de Toulouse Oncopole, Université de Toulouse 3 Paul Sabatier, Toulouse, France; 13 Department of Hemato Oncology, Seoul National University Hospital, Seoul, Republic of Korea; 14 Department of Hematology and Oncology, Okayama University Hospital, Okoyama, Japan; 15 Department of Internal Medicine, University of Fukui, Fukui, Japan; 16 Department of Hematology, Hokkaido University, Sapporo, Japan; 17 Department of Hematology, University of Tsukuba, Tsukuba, Japan; 18 Catholic Hematology Hospital, College of Medicine, The Catholic University of Korea, Seoul, Republic of Korea; 19 Astellas Pharma US, Inc., Northbrook, Illinois; 20 Sidney Kimmel Comprehensive Cancer Center, Johns Hopkins University, Baltimore, Maryland

**Keywords:** Acute myelogenous leukemia, *FLT3* mutation, FLT3 inhibitor, Hematopoietic stem cell, transplantation, Post-transplantation maintenance therapy

## Abstract

The fms-like tyrosine kinase 3 (FLT3) inhibitor gilteritinib improved the survival of patients with relapsed or refractory (R/R) *FLT3*-mutated acute myelogenous leukemia (AML) in the phase 3 ADMIRAL trial. In this study, we assessed survival and relapse rates of patients in the ADMIRAL trial who underwent hematopoietic stem cell transplantation (HSCT), as well as safety outcomes in patients who received post-transplantation gilteritinib maintenance therapy. ADMIRAL was a global phase 3 randomized controlled trial that enrolled adult patients with *FLT3*-mutated R/R AML Patients with R/R AML who harbored *FLT3* internal tandem duplication mutations in the juxtamembrane domain or D835/I836 point mutations in the tyrosine kinase domain were randomized (2:1) to gilteritinib (120 mg/day) or to preselected high- or low-intensity salvage chemotherapy (1 or 2 cycles). Patients in the gilteritinib arm who proceeded to HSCT could receive post-transplantation gilteritinib maintenance therapy if they were within 30 to 90 days post-transplantation and had achieved composite complete remission (CRc) with successful engraftment and no post-transplantation complications. Adverse events (AEs) during HSCT were recorded in the gilteritinib arm only. Survival outcomes and the cumulative incidence of relapse were assessed in patients who underwent HSCT during the trial. Treatment-emergent AEs were evaluated in patients who restarted gilteritinib as post-transplantation maintenance therapy. Patients in the gilteritinib arm underwent HSCT more frequently than those in the chemotherapy arm (26% [n = 64] versus 15% [n = 19]). For all transplantation recipients, 12- and 24-month overall survival (OS) rates were 68% and 47%, respectively. Despite a trend toward longer OS after pretransplantation CRc, post-transplantation survival was comparable in the 2 arms. Patients who resumed gilteritinib after HSCT had a low relapse rate after pretransplantation CRc (20%) or CR (0%). The most common AEs observed with post-transplantation gilteritinib therapy were increased alanine aminotransferase level (45%), pyrexia (43%), and diarrhea (40%); grade ≥3 AEs were related primarily to myelosuppression. The incidences of grade ≥III acute graft-versus-host disease and related mortality were low. Post-transplantation survival was similar across the 2 study arms in the ADMIRAL trial, but higher remission rates with gilteritinib facilitated receipt of HSCT. Gilteritinib as post-transplantation maintenance therapy had a stable safety and tolerability profile and was associated with low relapse rates. Taken together, these data support a preference for bridging therapy with gilteritinib over chemotherapy in transplantation-eligible patients.

## INTRODUCTION

Activating *fms-like tyrosine kinase 3* (*FLT3*) mutations in patients with acute myelogenous leukemia (AML) are common and associated with aggressive disease and poor survival [1,2]. Historically, internal tandem duplication mutations in *FLT3* (*FLT3*-ITD) were associated with frequent early relapse and short disease-free survival and overall survival (OS) after standard chemotherapy [2,3], especially in patients with a high *FLT3*-ITD allelic ratio [3]. Given the poor rates of second remission after standard salvage chemotherapy (SC) in relapsed or refractory (R/R) *FLT3*-mutated *(FLT3*^mut+^) AML, hematopoietic stem cell transplantation (HSCT) in first remission is generally recommended for eligible newly diagnosed patients harboring *FLT3-YYD* mutations [4], as well as patients with *FLT3* tyrosine kinase domain (*FLF3*-TKD) point mutations in the absence of *NPM1* co-mutations [5].

To improve outcomes in patients with *FLT3*^mut+^ AML, FLT3 inhibitors have been integrated into chemotherapy and transplantation algorithms. Adding the multikinase inhibitor midostaurin to intensive frontline chemotherapy regimens was found to improve OS in patients with newly diagnosed *FLT3*^mut+^ AML [6]. Studies performed in first remission show that sorafenib lowers relapse rates and may improve survival when given as post-transplantation maintenance therapy in patients with *FLT3*-ITD mutations [7,8]. Older FLT3 inhibitors, such as midostaurin, have limited efficacy as single-agent therapy in patients with *FLT3*^mut+^ AML, possibly related to their relatively limited potency in vivo [9,10]. More recently developed FLT3 inhibitors, such as gilteritinib, have shown improved in vivo potency, significant single-agent clinical activity in *FLT3*^mut+^ R/R AML, and favorable tolerability at clinically active doses [11,12].

The phase 3 ADMIRAL trial showed that gilteritinib improved the survival of patients with R/R AML and an activating *FLT3* mutation compared with SC [13], leading to its regulatory approval for this indication [14]. Among patients enrolled in ADMIRAL, 19% had relapsed after a prior HSCT and 40% were considered ineligible for intensive SC [13]. A greater proportion of patients in the gilteritinib arm (26%) than in the SC arm (15%) proceeded to HSCT. Notably, the study design allowed gilteritinib arm patients to resume gilteritinib therapy after HSCT if they achieved composite complete remission (CRc) and had stable engraftment without serious post-transplantation complications [13].

The improvement in OS observed with gilteritinib in ADMIRAL was maintained when the results were censored at the time of HSCT, and improved survival also was observed in gilteritinib arm patients eligible for intensive SC at study entry. However, a detailed analysis of post-transplantation outcomes from the ADMIRAL trial had not been conducted. Therefore, we performed a post hoc analysis to evaluate outcomes in patients who underwent HSCT in the ADMIRAL trial with respect to OS, pretransplantation response, and post-transplantation relapse. The impact of post-transplantation gilteritinib maintenance therapy on OS and the safety profile of gilteritinib maintenance therapy were assessed as well.

## METHODS

### Statement of Ethics

The study protocol for ADMIRAL (CIinicalTrials.gov identifier NCT02421939) was approved by an independent Ethics Committee or Institutional Review Board at each participating site. All patients provided written informed consent at the time of enrollment.

### Patient Population and Study Design

Enrolled patients were age ≥18 years and in untreated first relapse after achieving complete remission (CR) with or without complete hematologic or platelet recovery with initial induction therapy or were refractory to initial induction therapy. All patients had a confirmed *FLT3*-ITD mutation or *FLT3*-TKD D835/I836 point mutation based on central laboratory testing (LeukoStrat CDx *FLT3* Mutation Assay; Invivoscribe, San Diego, CA); local laboratory testing for *FLT3* mutations was permitted in cases of aggressive disease. Complete inclusion/exclusion criteria were outlined in the primary publication [13]. Patients were randomized 2:1 to receive 120 mg/day gilteritinib or 1 of 4 high- or low-intensity SC regimens selected prior to randomization. High-intensity SC was administered for 1 to 2 cycles. Gilteritinib or low-intensity chemotherapy was administered in continuous 28-day cycles until a treatment discontinuation criterion was met.

### Post-Transplantation Administration of Gilteritinib

For patients who proceeded to HSCT, gilteritinib therapy was stopped prior to beginning the conditioning regimen for HSCT. Patients could resume gilteritinib after HSCT if they achieved CRc and were between 30 and 90 days post-transplantation with successful engraftment (ie, absolute neutrophil count [ANC] ≥500/mm^3^ and platelet count ≥2000/mm^3^ without transfusions) without grade ≥II acute graft-versus-host disease (GVHD). Adverse events (AEs) associated with HSCT were not systematically collected until gilteritinib was restarted. Patients who resumed gilteritinib after HSCT were required per protocol to undergo routine bone marrow evaluation, generally every 3 months, to document ongoing response.

### Data Analyses and Assessments

Response and survival outcomes were assessed in all patients who underwent HSCT. An analysis of a subset of gilteritinib arm patients who underwent HSCT and were without relapse for 60 days after HSCT was also performed. Response was assessed using modified International Working Group criteria (Supplementary Table S1) [15]. CRc was defined as the sum of patients who achieved CR, CR with incomplete hematologic recovery (CRi), and CR with incomplete platelet recovery (CRp); CRh was defined as CR with partial hematologic recovery (Supplementary Table S1). OS was landmarked to the date of HSCT or assessed using a time-dependent Mantel-Byar analysis [16–18] that avoids bias due to variability in the time to transplantation [17]. At randomization, Mantel-Byar analysis assigns all patients to the no-transplantation risk cohort; patients from the no-transplantation cohort are censored and enter the transplantation risk cohort at the time of HSCT. Survival outcomes were compared using the log-rank test as described previously [18]. AEs were assessed using National Cancer Institute’s Common Terminology Criteria for Adverse Events version 4.03 criteria.

### Statistical Analyses

Descriptive statistics were used to assess continuous variables. Categorical data were reported as frequency and percentage. Hazard ratios (HRs) and supporting confidence intervals (CIs) were used to determine differences in OS between groups. Reported *P* values were based on the Mantel-Byar test with continuity correction. Because the statistical analysis plan did not include provisions for multiplicity correction with respect to evaluation of secondary outcomes or subgroup analyses, these results were reported as point estimates with 95% CIs. Statistical analyses were performed with SAS version 9.3 or higher software (SAS Institute, Cary, NC).

## RESULTS

### Patient Disposition and Baseline Characteristics

**Figure 1.**
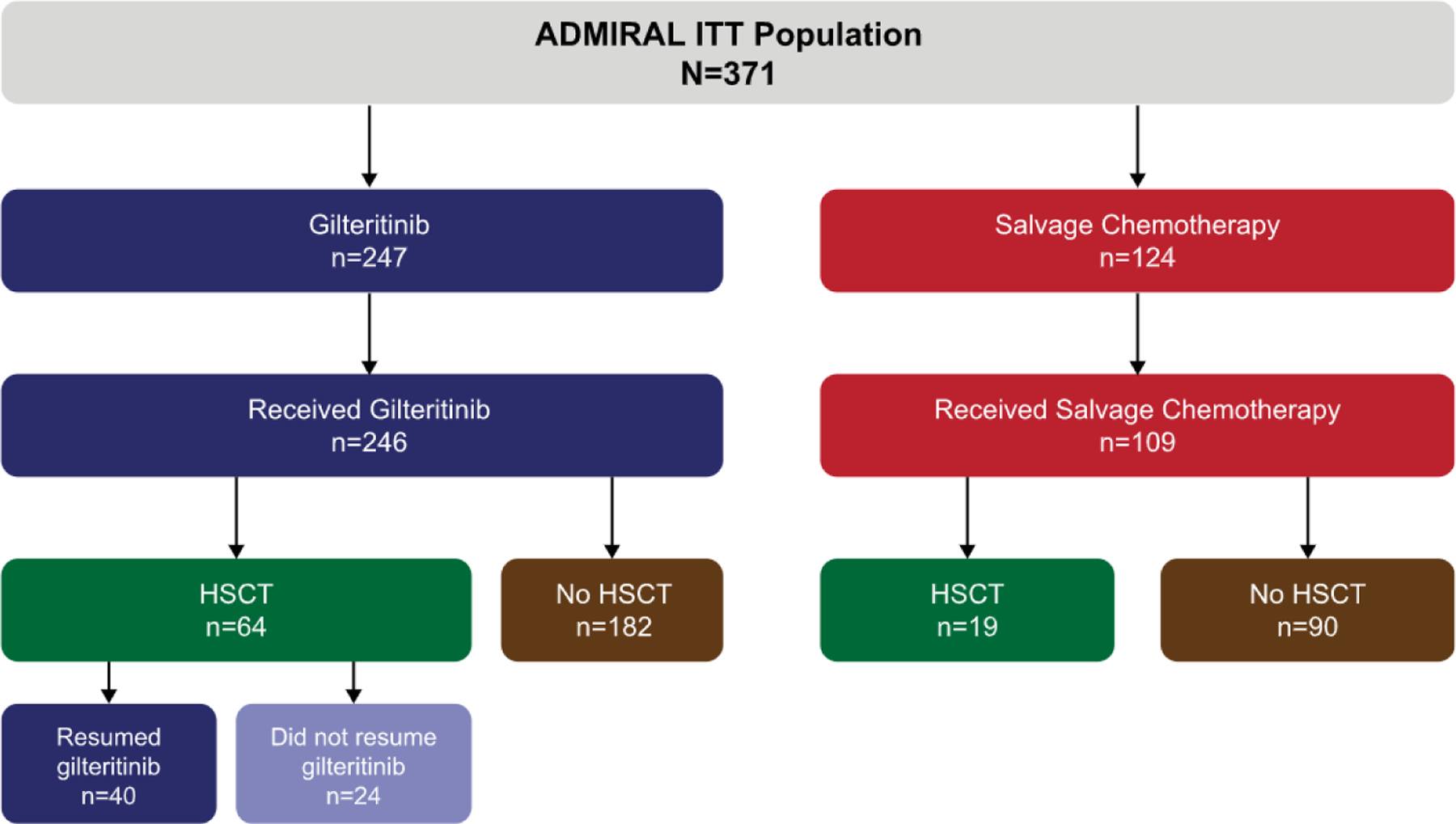
Patient disposition. ITT indicates intention to treat.

As of the data cutoff date of September 20, 2020, 83 patients (gilteritinib arm, n = 64; SC arm, n = 19) in ADMIRAL had undergone HSCT (Figure 1). Of the 64 gilteritinib arm patients who underwent HSCT, 40 resumed gilteritinib as post-transplantation maintenance therapy. Nine gilteritinib arm patients received a post-transplantation FLT3 inhibitor in the nonmaintenance setting; 7 of these 9 patients (78%) received a FLT3 inhibitor after relapse (sorafenib, n = 4; midostaurin, n = 2; gilteritinib, n = 1). The remaining 2 gilteritinib arm patients did not achieve post-transplantation CRc and subsequently received sorafenib. Of the 19 patients in the SC arm who underwent HSCT, 2 (11%) received sorafenib after achieving remission; however, 1 of these patients subsequently relapsed and then received gilteritinib.

Demographic and baseline characteristics of the patients who underwent HSCT are shown in Table 1. Of 371 patients enrolled in ADMIRAL, 74 (20%) had undergone prior HSCT. During the trial, 75 of 371 patients (20%) underwent a first transplantation and 8 (2%) underwent a second transplantation. Most patients who underwent HSCT were age <65 years (89%; n = 74 of 83) and had been preselected for high-intensity chemotherapy (87%; n = 72 of 83). The median allelic ratio of *FLT3-*ITD to wild-type *FLT3* for the study population was .77, with allelic ratios ≥.77 defined as high and those <.77 defined as low. The proportion of patients with a high *FLT3*-ITD allelic ratio (ie, ≥.77) at baseline was lower in the HSCT group compared with the non-HSCT group (36% versus 48%). Forty of the 64 gilteritinib arm patients (63%) who underwent HSCT resumed gilteritinib after HSCT, for a median of 295 days (range, 1 to 1505 days). Fifty-three gilteritinib arm patients were without relapse for 60 days after HSCT; 36 (68%) of these patients resumed gilteritinib after HSCT. Baseline demographic and disease characteristics and prior treatment characteristics were generally similar between patients who resumed gilteritinib after HSCT and those who did not (Supplementary Table S2).

Detailed transplantation characteristics were available for 55 gilteritinib arm patients who underwent HSCT (Table 2); transplantation characteristics were not captured for the SC arm. Most of these patients (71%; n = 39) received conditioning regimens containing purine analogs combined with single or double alkylators. The median time to transplantation from the first dose of study treatment was 3.5 months (range, 1.3 to 12.2 months) in the gilteritinib arm and 2.4 months (range, .4 to 5.5 months) in the SC arm. AML Hematopoietic Cell Transplantation Comorbidity Index values [19] were not determined.

**Figure 2.**
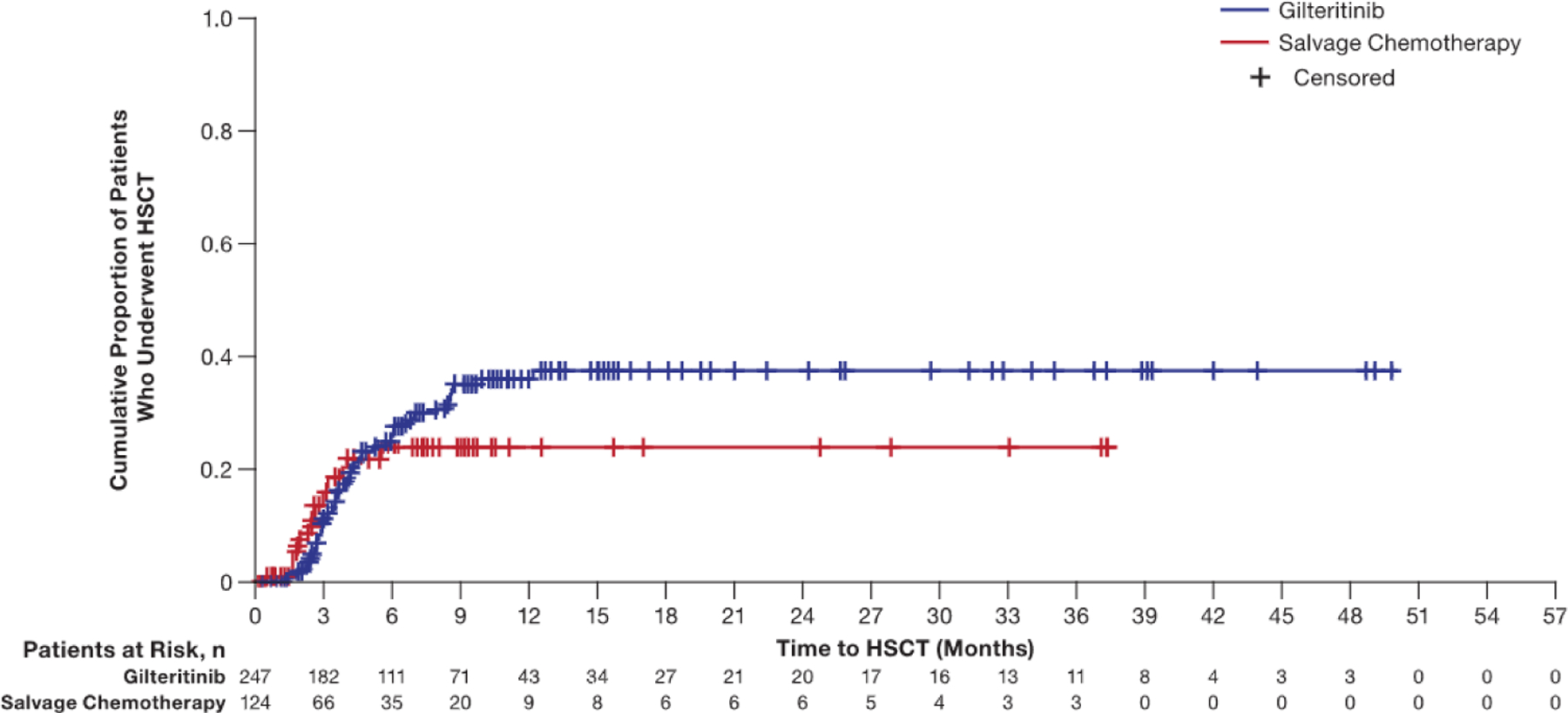
Cumulative incidence of transplantation.

Nearly all patients in the SC arm underwent HSCT during the first 6 months of treatment, whereas in the gilteritinib arm, HSCTs were performed over a 12-month period (Figure 2). The median time to reach an ANC >500/mm^3^ and a platelet level ≥20,000/mm^3^ with transfusion independence in patients who resumed gilteritinib therapy was transplant day +51 (interquartile range, day +39 to day +74). The majority (87%; n = 48 of 55) of these patients remained in remission after HSCT; the median duration of CR or CRc had not been reached. Data related to loss of chimerism or to primary or secondary engraftment were not available.

### OS by Transplantation and Remission Status

**Figure 3.**
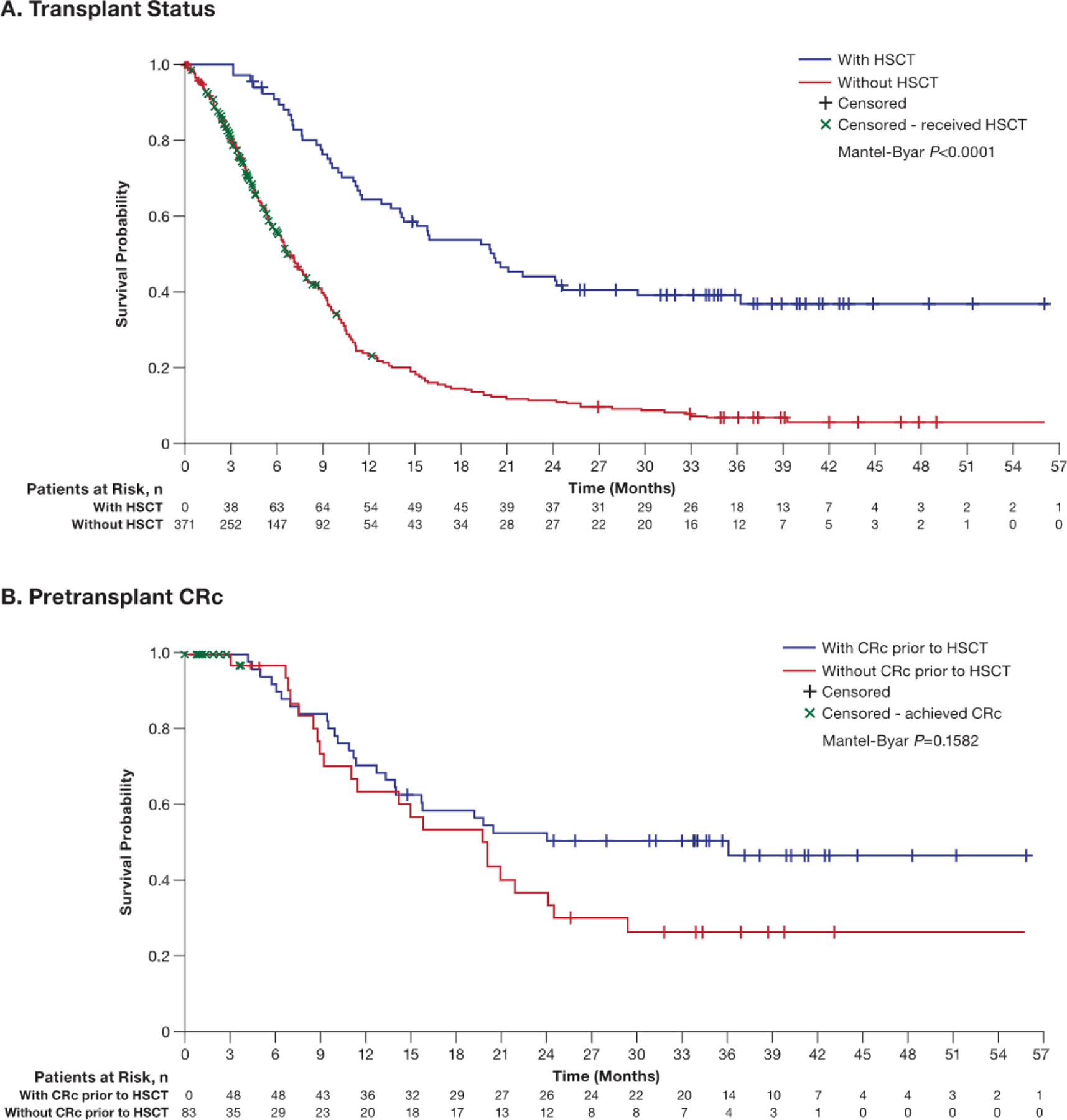
OS by transplantation type and achievement of pretransplantation CRc: pooled analysis of the gilteritinib and SC arms.^a a^Graphical representations of Mantel-Byar estimates differ from typical Kaplan-Meier estimates, as the components of the curves shown are not predefined at time 0 and change throughout the displayed time.

The median follow-up was similar in the HSCT and non-HSCT groups (35.9 months [95% CI, 34.0 to 39.9 months] versus 37.4 months [95% CI, 35.1 to 42.0 months]). The median OS was 20.2 months (95% CI, 14.1 to 36.2 months) in all patients who underwent HSCT (n = 83) and 6.8 months (95% CI, 6.1 to 7.9 months) in patients who did not undergo HSCT. As shown in Figure 3A, the median OS by Mantel-Byar analysis was significantly longer among patients who underwent HSCT than in patients who did not undergo HSCT (20.2 months [95% CI, 14.1 to 36.2 months] versus 6.8 months [95% CI, 6.1 to 7.9 months]; *P* < .0001). Respective rates of OS at 12 and 24 months were 65% and 44% in patients who underwent HSCT and 23% and 12% in patients without HSCT. OS assessed by Mantel-Byar analysis in patients who achieved pretransplantation CRc and those who did not is shown in Figure 3B.

### Post-Transplantation Survival and Relapse

**Figure 4.**
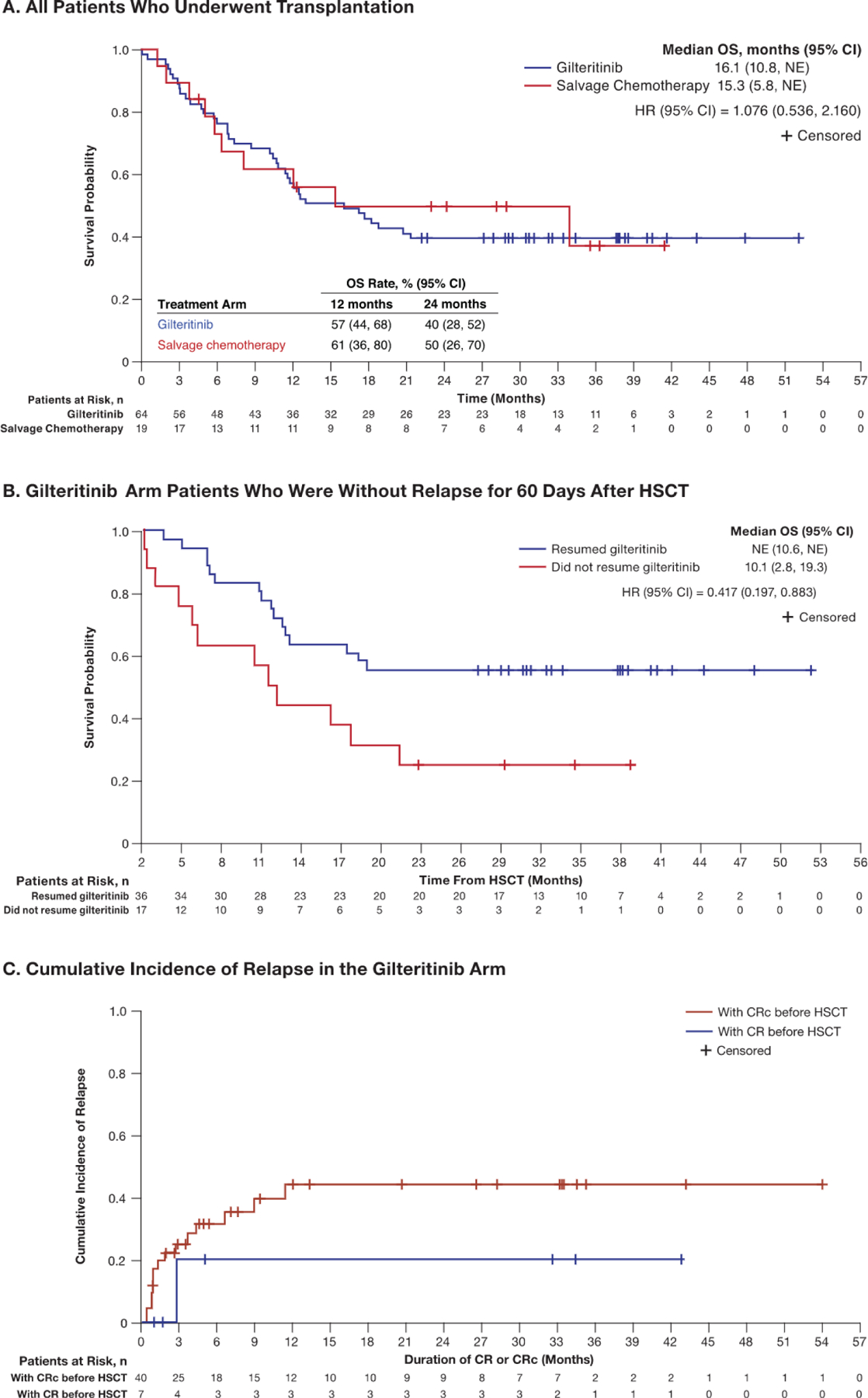
Post-transplantation OS in patients with *FLT3*^mut+^ R/R AML landmarked to the date of transplantation. NE indicates not evaluable.

The median OS landmarked to the date of HSCT was 16.1 months in the gilteritinib arm and 15.3 months in the SC arm (HR, 1.076; 95% CI, .536 to 2.160) (Figure 4A). The OS rates at 12 and 24 months in the gilteritinib arm were 57% and 40%, respectively; corresponding OS rates in the SC arm were 62% and 50%.

Among gilteritinib arm patients who were alive and without relapse for 60 days after HSCT, the median OS land-marked from post-transplantation day 60 had not been reached at the time of data cutoff in patients who resumed gilteritinib after HSCT (95% CI, 10.6 months to not reached). The median OS in patients who did not resume gilteritinib therapy was 10.1 months (95% CI, 2.8 to 19.3 months) (Figure 4B).

The reasons for not resuming gilteritinib after HSCT in 17 patients included progressive disease in 5, physician decision due to failure to meet protocol-defined criteria for restarting gilteritinib in 4, relapse in 2, lack of efficacy in 2, AEs in 1, GVHD in 1, CRc for >90 days after HSCT in 1, and lack of post-transplantation bone marrow, ANC, or platelet assessment and subsequent platelet transfusion in 1.

### Pretransplantation Response

Although patients were not required to be in CRc to undergo HSCT, high pretransplantation remission rates were observed in both study arms (Table 3). Of the 64 gilteritinib arm patients who underwent HSCT, 40 (63%) achieved pre-transplantation CRc and 24 (38%) did not (partial remission, 22% [n = 14]; no response, 16% [n=10]). Of 19 patients who underwent HSCT in the SC arm, 11 (58%) achieved pretransplantation CRc and 8 (42%) did not (partial remission, 5% [n = 1]; no response, 16% [n = 3]; not evaluable, 21% [n = 4]). Patients who did not restart gilteritinib more frequently underwent HSCT without CRc. Nine gilteritinib arm patients who underwent HSCT had been preselected for low-intensity chemotherapy, and all 9 achieved pretransplantation CRc (CR, 67%; CRi, 33%).

### OS by Pretransplantation Treatment Response

Among the patients who underwent HSCT, no significant difference in OS (landmarked to the date of HSCT) was observed between the treatment arms among patients who achieved pretransplantation CRc and those who did not (Supplementary Figure S1A,B), as well as patients who achieved pretransplantation CR/CRh (Supplementary Figure S2). The median OS for patients who did not undergo HSCT after achieving remission was shorter in both treatment arms (CRc: gilteritinib arm, 10.7 months; SC arm, 9.3 months; CR/CRh: gilteritinib arm, 15.8 months; SC arm, 9.3 months) compared with the median OS for the corresponding HSCT recipient groups (CRc: gilteritinib arm, not reached; SC arm, 36.2 months; CR/CRh: gilteritinib arm, not reached; SC arm, 36.2 months).

### Post-Transplantation Relapse

Of the 64 gilteritinib arm patients who underwent HSCT, 52 (81%) achieved CRc either before or after transplantation; 36 of these 52 (69%) patients resumed gilteritinib after HSCT. Eight of these 36 patients relapsed; the median time to relapse was 6.6 months (range, 4.8 to 15.2 months) from the date of HSCT. The 17 patients who did not resume gilteritinib therapy after transplantation were not required to undergo regular bone marrow evaluation per protocol and underwent follow-up for OS. Seven of these 17 patients had achieved pretransplantation CRc (CR, n = 1; CRi, n = 5; CRp, n = 1); 2 of these 7 patients had relapsed before HSCT.

The cumulative incidence of relapse in gilteritinib arm patients from the time of achieving pretransplantation CR or CRc is shown in Figure 4C. Most relapses occurred within the first 12 months after achievement of CRc. The cumulative relapse rate at 12 months in gilteritinib arm patients who underwent HSCT was 20% after achieving pretransplantation CR (n = 7) and 45% after achieving pretransplantation CRc (n = 40) and remained unchanged at 24 months post-transplantation.

Among the patients who resumed gilteritinib after HSCT and had achieved a pretransplantation CR (n = 4) or CRc (n = 20), the cumulative relapse rates were 0% and 19%, respectively, at both 12 and 24 months. Among 53 gilteritinib arm patients without relapse for 60 days post-HSCT, the pretransplantation rate of CRc was higher in patients who resumed gilteritinib after HSCT compared with those who did not resume gilteritinib (72% versus 41%) (Table 4).

Of the 40 patients who resumed gilteritinib after HSCT, 29 had discontinued treatment at the time of data cutoff and 11 continued to receive gilteritinib. The most common reasons for discontinuation were death (24%; n = 7 of 29), relapse (21%; n = 6 of 29), AEs (21%; n = 6 of 29), and physician decision (17%; n = 5 of 29).

### Drug Exposure and Post-Transplantation Adverse Events after Restart of Gilteritinib Therapy

**Figure 5.**
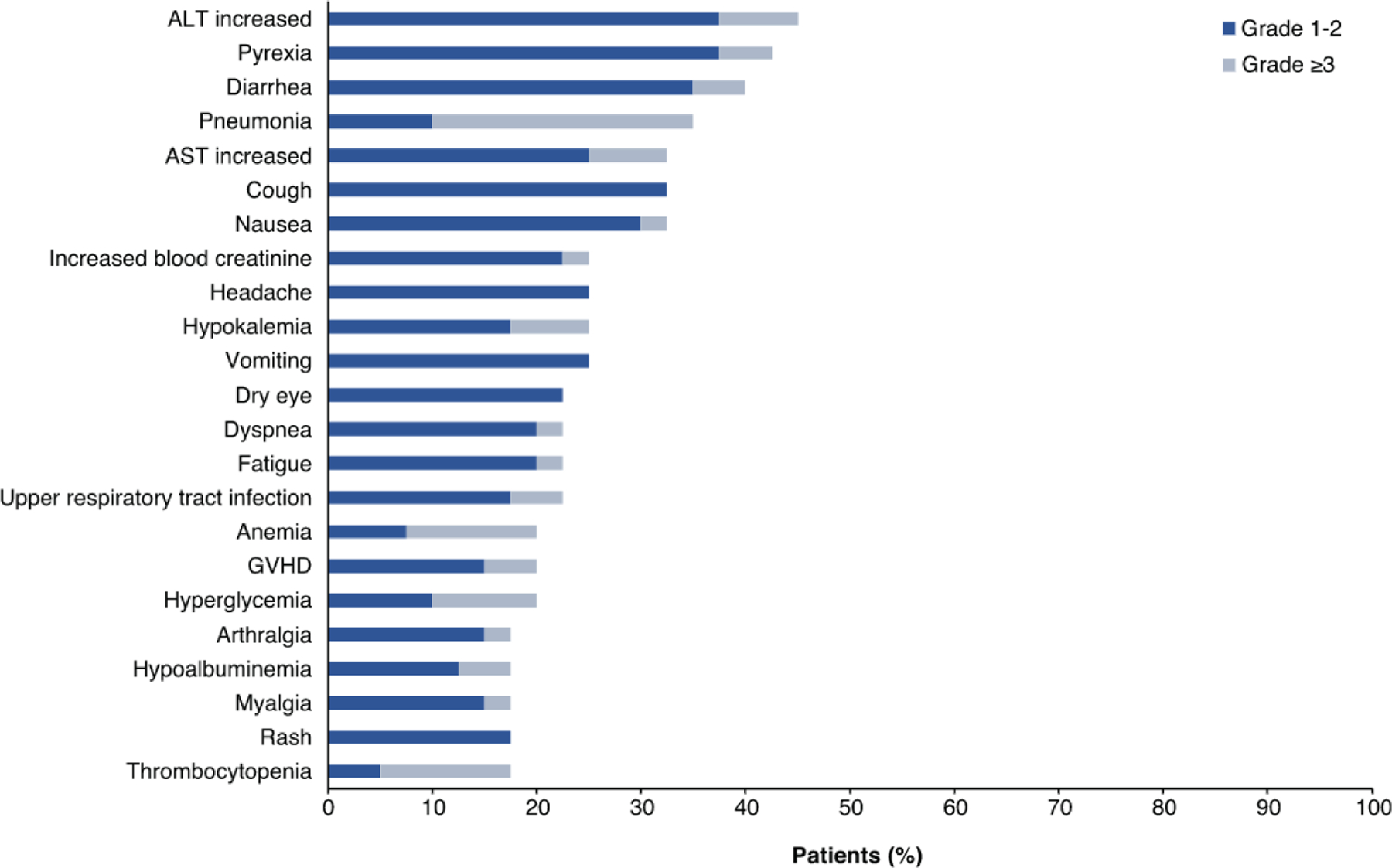
Incidence of post-transplantation AEs after restart of gilteritinib therapy.^a a^All AEs during restart of gilteritinib and within 30 days from the last study treatment were reported. AST indicates aspartate aminotransferase.

In the patients who resumed gilteritinib after HSCT, the median dose of gilteritinib was 120 mg (range, 40 to 200), and the median duration of posttransplant gilteritinib maintenance therapy was 258.5 days (IQR, 51.5 to 823 days). The rate of grade ≥II acute GVHD after the restart of gilteritinib was 33% (n = 13 of 40). Among the patients with grade ≥III acute GVHD after resuming gilteritinib therapy (10%; n = 4 of 40), 1 case of fatal acute gastrointestinal GVHD was observed. The most frequent AEs of any grade occurring after resuming gilteritinib were increased alanine aminotransferase (ALT) level (45%), pyrexia (43%), and diarrhea (40%) (Figure 5). Common grade ≥3 AEs were pneumonia (25%), anemia (13%), and thrombocytopenia (13%).

The most common grade ≥3 AEs of interest were increased liver transaminase (ALT or aspartate aminotransferase) level (8%) (Supplementary Figure S3). Grade ≥3 prolonged QT interval occurred in 1 patient; other grade ≥3 cardiac AEs of interest included ventricular tachycardia, cardiac arrest, and cardiorespiratory arrest (all n = 1). Grade ≥3 AEs related to gastrointestinal hemorrhage were not reported in any patients. Six patients died during the post-transplantation period due to acute GVHD in the intestine (n = 1), cardiac arrest (n = 1), bacterial sepsis (n = l), respiratory syncytial virus infection and respiratory tract fungal infection (n = l), pneumothorax and pulmonary embolism (n = l), and an unknown cause (n = l); all deaths were unrelated to treatment.

Seven of the 40 patients (18%) who resumed gilteritinib after HSCT experienced AEs that led to dosage reductions (1 each with grade 4 thrombocytopenia and neutropenia, grade 3 hypokalemia, grade 1 peripheral edema and grade 3 weight gain, grade 2 pleural effusion, grade 3/4 increased blood creatine phosphokinase, grade 1 pleural thickening, and grade 2 increased ALT). AEs leading to dosage interruptions occurred in 19 of the 40 patients (48%) who resumed gilteritinib after HSCT. Overall, 14 of 40 patients (35%) experienced grade ≥3 AEs leading to dosage interruptions; 6 patients (15%) experienced drug-related grade ≥3 AEs (pancytopenia, increased blood lactate dehydrogenase, hypokalemia, dermatomyositis, respiratory failure, and thrombocytopenia).

## DISCUSSION

The emergence of FLT3-targeted therapies administered in frontline or R/R settings enables patients with *FLT3*^mut+^ AML to achieve durable remission and serves as a bridge to HSCT. Beyond a higher response rate than SC, gilteritinib offers other potential benefits to patients with R/R AML in combination with HSCT. The lower toxicity of gilteritinib compared with intensive SC facilitates transplantation by reducing the likelihood of unresolved toxicities of chemotherapy, which may improve the tolerability of the preparative regimen and reduce other transplantation-related complications. Maintenance therapy with gilteritinib also may allow patients to remain in remission after HSCT [21].

This post hoc analysis shows that bridging treatment with either gilteritinib or SC led to equivalent post-transplantation survival. Patients who resumed gilteritinib after HSCT had low relapse rates and longer OS compared with those who did not. However, we caution against interpretation of any definitive treatment effects of gilteritinib maintenance therapy from ADMIRAL. The number of patients who received gilteritinib was small and a pretransplantation response of CRc was more common among patients who restarted gilteritinib, which might have contributed to the observed differences in survival. Additionally, neither secondary randomization to determine definitive treatment effects of maintenance therapy nor routine bone marrow evaluation for gilteritinib arm patients who did not resume gilteritinib were incorporated into the study design. However, patients who resumed gilteritinib experienced no new safety signals during the post-transplantation period. Grade ≥3 AEs in patients receiving post-transplantation gilteritinib were related primarily to myelosuppression; a low risk for grade ≥3 AEs related to hepatic dysfunction and cardiac events persisted. Rates of grade ≥3 GVHD after gilteritinib resumption and GVHD-related mortality were low.

Post-transplantation maintenance therapy with FLT3 inhibitors in patients with AML remains off-label in the United States but is a growing area of interest. The randomized, placebo-controlled, double-blind SORMAIN trial showed that up to ~2 years of post-transplantation sorafenib therapy in patients with *FLT3*-ITD–positive AML significantly reduced the risk of post-transplantation relapse or death compared with placebo (HR for relapse or death, .39; 95% CI, .18 to .85; *P* = .013) [7]. Additionally, in an open-label randomized trial of sorafenib versus no post-transplantation maintenance therapy in patients with *FLT3*-ITD–positive AML, Xuan et al. [8] demonstrated that post-transplantation sorafenib therapy was associated with significantly reduced 1-year cumulative incidence of relapse (HR, .25; 95% CI, .11 to .57; *P*=.0010) and superior OS (HR, .48; 95% CI, .27 to .86; *P*=.012) [8]. In a single-arm study, post-transplantation midostaurin use also was associated with a significantly improved OS (multivariable *P*=.01 versus no midostaurin) and event-free survival (multi-variable *P*=.004 versus no midostaurin) versus historical controls with *FLT3*-ITD–positive AML in first CR/CRi [22]. Maziarz et al. [23] reported lower post-transplantation relapse rates among patients randomized to receive 1 year of single-agent maintenance therapy with midostaurin compared with those receiving usual care (11% versus 24%) [23].

The aforementioned studies mainly examined HSCT in first remission, when the risk of relapse may be lower and the risk/benefit ratio for post-transplantation maintenance therapy may differ from that in the R/R AML setting of ADMIRAL. The phase 3 QuANTUM-R trial of quizartinib versus SC in patients with R/R *FLT3*-ITD–positive AML had a similar design as ADMIRAL. Patients in QuANTUM-R who received quizartinib maintenance therapy after pretransplantation CRc had longer OS than those who did not receive post-transplantation quizartinib (27.1 months versus 5.4 months) [24]. Rates of OS at 1 year and 2 years post-transplantation also were markedly higher in patients who resumed quizartinib after HSCT compared with those who did not (1 year, 77% versus 12%; 2 years, 64% vs 12%) [24]. Given the similar study design as ADMIRAL, the effect of quizartinib maintenance therapy on survival in QuANTUM-R also should be interpreted with caution.

As is typical of secondary analyses, our study has several limitations. The small number of patients who underwent HSCT during the trial precluded rigorous statistical comparisons between HSCT and non-HSCT groups. In addition, our statistical analyses were not adjusted for multiple comparisons. Because patients who received high-intensity SC discontinued study treatment after 1 or 2 treatment cycles, long-term follow-up was restricted to a very small number of SC arm patients, and post-transplantation AEs were not rigorously monitored. The gilteritinib arm patients did not participate in routine study visits during the transplantation period prior to restarting gilteritinib, and thus it is plausible that patients who restarted gilteritinib therapy likely experienced fewer transplantation-associated toxicities than patients who did not restart gilteritinib, and cross-arm comparisons of transplantation-associated AEs were not possible. Patients who underwent HSCT were younger (<65 years) and considered eligible for high-intensity chemotherapy, which likely predisposed them to better survival outcomes. Post-transplantation use of another FLT3 inhibitor also might have affected OS in both arms. Furthermore, a smaller proportion of patients who underwent HSCT had a high *FLT3*-ITD allelic ratio compared with patients who did not undergo HSCT. Finally, pretransplantation assessments of measurable residual disease were not conducted before HSCT, which could have significantly influenced post-transplantation outcomes.

Our analysis demonstrates that patients with *FLT3*^mut+^ R/R AML derive a significant survival benefit with HSCT, but survival was similar regardless of whether gilteritinib or SC was used as a bridge to transplantation [13]. Because response and transplantation rates were higher in the gilteritinib arm compared with the SC arm and toxicity was lower in the gilteritinib arm, we conclude that gilteritinib is the preferred salvage treatment for transplantation-eligible patients with R/R *FLT3*^mut+^ AML. The safety and tolerability of gilteritinib appear stable in the post-transplantation setting. Although late relapse was quite rare in our cohort, our study can neither definitively determine the merits of maintenance on survival nor clarify the optimal duration of post-transplantation gilteritinib. An ongoing, phase 3, placebo-controlled study (Clinical-Trials.gov identifier NCT02997202) will evaluate the benefit of long-term post-transplantation gilteritinib therapy in first morphologic CR and is anticipated to inform therapy for patients with advanced AML undergoing HSCT.

## Supplementary Material

Supplemental file

## Figures and Tables

**Table 1:** Baseline Characteristics of Patients With *FLT3*^mut+^ R/R AML Who Underwent HSCT

Characteristic	HSCT		No HSCT	
	Gilteritinib (n = 64)	SC (n = 19)	Gilteritinib (n = 183)	SC(n = 105)
Age, yr, median (range)	54.5 (21–71)	51.0 (19–67)	66 (20–84)	63 (20–85)
Age <65 yr, n (%)	56 (88)	18 (95)	85 (46)	57 (54)
Age ≥65 yr, n (%)	8 (13)	1 (5)	98 (54)	48 (46)
Female sex, n (%)	33 (52)	10 (53)	98 (54)	60 (57)
ECOG performance status, n (%)				
0–1	63 (98)	16 (84)	143 (78)	89 (85)
≥2	1 (2)	3 (16)	40 (22)	16 (15)
*FLT3* mutation type, n (%)*				
* FLT3*-ITD	55 (86)	16 (84)	160 (87)	97 (92)
* FLT3-*TKD	6 (9)	2 (11)	15 (8)	8 (8)
* FLT3-*ITD and *FLT3*-TKD	3 (5)	0	4 (2)	0
Unknown/missing	0	1 (5)	4 (2)	0
Cytogenetic risk status, n (%)				
Favorable	1 (2)	0	3 (2)	1 (<1)
Intermediate	52 (81)	12 (63)	130 (71)	77 (73)
Unfavorable	2 (3)	5 (26)	24 (13)	6 (6)
Other/unknown	9 (14)	2 (11)	26 (14)	21 (20)
Preselected chemotherapy by IRT, n (%)				
High intensity	55 (86)	17 (90)	94 (51)	58 (55)
Low intensity	9 (14)	2 (11)	89 (49)	47 (45)
Response to first-line therapy by IRT, n (%)				
Relapsed ≤6 mo after allogeneic HSCT	3 (5)	0	28 (15)	17 (16)
Relapsed ≤6 mo after CRc without HSCT	20 (31)	5 (26)	47 (26)	29 (28)
Relapsed >6 mo after allogeneic HSCT	2 (3)	3 (16)	15 (8)	5 (5)
Relapsed >6 mo after CRc without HSCT	5 (8)	2 (11)	29 (16)	15 (14)
Primary refractory without HSCT	34 (53)	9 (47)	64 (35)	39 (37)^†^
Prior anthracyclines, n (%)				
Yes	63 (98)	18 (95)	142 (78)	88 (84)
No	1 (2)	1 (5)	41 (22)	17 (16)
Prior TKI therapy, n (%)				
Midostaurin	3 (5)	0	11 (6)	9 (9)
Sorafenib	2 (3)	0	17 (9)	6 (6)
Prior HSCT, n (%)				
Yes	5 (8)	3 (16)	43 (24)	23 (22)^†^
No	59 (92)	16 (84)	140 (77)	82 (78)
*FL73*-ITD allelic ratio, n (%)				
High	23 (36)	7 (37)	86 (47)	53 (50)
Low	35 (55)	9 (47)	78 (43)	44 (42)
Missing	6 (9)	3 (16)	19 (10)	8 (8)
Common co-mutations, n (%)				
* NPM1*	29 (45)	5 (26)	86 (49)	53 (51)
* DNMT3A*	21 (33)	4 (21)	54 (31)	36 (35)
* NPM1* and *DNMT3A*	17 (27)	3 (16)	38 (22)	28 (27)
* WT1* ^‡^	16 (25)	3 (16)	29 (16)	17 (16)
* IDH1/IDH2* ^‡^	5 (8)	3 (16)	33 (19)	15 (14)
No common co-mutations, n (%)^§^	17 (27)	10 (53)	52 (28)	31 (30)

ECOG indicates Eastern Cooperative Oncology Group; IRT, interactive response technology; ITT, intention to treat; TKI, tyrosine kinase inhibitor.

* All patients had a *FLT3* mutation confirmed by central laboratory testing, except for 1 patient who had a *FLT3*-TKD mutation confirmed by local laboratory testing.

† Includes 1 patient who had prior HSCT 150 days before randomization but was classified as primary refractory without HSCT by IRT; the patient discontinued treatment 5 days after randomization on the investigators’ decision.

‡ *WT1* and *IDH1/IDH2* mutations were not mutually exclusive of mutations in *NPM1* and *DNMT3A*.

§ Limited to *NPM1, DNMT3A, WT1 ,IDH1,* and *IDH2*.

**Table 2:** Transplantation Characteristics of Patients in the Gilteritinib Arm (N = 55)*

Characteristic	Value
Graft type, n (%)	
Allogeneic	54 (98)
Autologous	1 (2)
Donor type, n (%)	
Related	28 (51)
Matched	19 (34)
Partially matched	9 (16)
Unrelated	26 (47)
Matched	16 (29)
Partially matched	10 (18)
Conditioning regimen, n (%)	
Busulfan + cyclophosphamide	5 (9)
Cyclophosphamide + TBI ± cytarabine	4 (7)
High-dose TBI ± other agents	2 (4)
Purine analog + double alkylator ± low-dose TBI	15 (27)
Purine analog + single alkylator ± low-dose TBI	24 (44)
Other	5 (9)
Transplantation outcome, n (%)	
Continued CR	48 (87)
Relapse	5 (9)
Engraftment failure	1 (2)
Rejection	0
Missing	1 (2)

TBI indicates total body irradiation.

* Represents patients for whom complete details related to transplantation were included in the case report form; details of transplantation were not reported for 9 patients. Low-dose TBI includes doses of <800 cGy for fractionated doses and ≤500 cGy for a single dose; all others were high-dose TBI [20].

**Table 3:** Pretransplantation Response in Patients with *FLT3*^mut+^ R/R AML Who Underwent HSCT

Response Parameter	Gilteritinib Arm (n = 64)	SC Arm (n = 19)
CR	7 (11)	6 (32)
CRi	26 (41)	5 (26)
CRp	7 (11)	0
CRh	9 (14)	3 (16)
**CRc** *	**40 (63)**	**11 (58)**
**CR/CRh**	**16 (25)**	**9 (47)**
PR	14 (22)	1 (5)
NR	10 (16)	3 (16)
NE	0	4 (21)

Bold type indicates aggregate response rate.

PR indicates partial remission; NE, not evaluable; NR, no response.

* Defined as the sum of patients who achieved CR, CRi, and CRp.

**Table 4:** Pretransplantation Response in Gilteritinib-Treated Patients Who Were without Relapse for 60 Days after HSCT

Response Parameter	Resumed Gilteritinib (n = 36)	Did Not Resume Gilteritinib (n = 17)
CR	4 (11)	1 (6)
CRi	17 (47)	5 (29)
CRp	5 (14)	1 (6)
CRh	7 (19)	0
**CRc** *	**26 (72)**	**7 (41)**
**CR/CRh**	**11 (31)**	**1 (6)**
PR	7 (19)	5 (29)
NR	3 (8)	5 (29)

Bold type indicates aggregate response rate.

* Defined as the sum of patients who achieved CR, CRi, and CRp.

## Data Availability

Researchers may request access to anonymized participant-level data, trial-level data, and protocols from Astellas-sponsored clinical trials at www.clinicalstudydatarequest.com. For the Astellas criteria on data sharing, see https://clinicalstudydatarequest.com/Study-Sponsors/Study-Sponsors-Astellas.aspx.
