# Supplementary material for: Outcomes in Patients with FLT3-Mutated Relapsed/ Refractory Acute Myelogenous Leukemia Who Underwent Transplantation in the Phase 3 ADMIRAL Trial of Gilteritinib versus Salvage Chemotherapy
Source: Transplant Cell Ther. Author manuscript; Available in PMC 2023 May 17. (PMC10189888; doi:10.1016/j.jtct.2022.12.006)
Supplement: Supplemental file [file NIHMS1875976-supplement-Supplemental_file.docx]

**SUPPLEMENTARY INFORMATION**

**Table S1. Definitions of Response Parameters**

| **Response Parameter** | **Definition** |
| --- | --- |
| **Complete remission (CR)** | Presence of regenerating hematopoietic cells in the bone marrow and achievement of a morphologic leukemia-free state with absolute neutrophil count (ANC) ≥1 × 10^9^/L, platelet count ≥100 × 10^9^/L, normal bone marrow differential with <5% blasts, and red blood cell (RBC)/platelet transfusion independence with no evidence of extramedullary leukemia. |
| **Complete remission with partial hematologic recovery (CRh)** | Bone marrow blasts <5% with partial hematologic recovery defined as ANC ≥0.5 × 10^9^/L and platelet count ≥50 × 10^9^/L, with no evidence of extramedullary leukemia and cannot be classified as CR. |
| **Complete remission with incomplete platelet recovery (CRp)** | Achievement of all CR criteria except for platelet recovery (platelet count <100 × 10^9^/L). |
| **Complete remission with incomplete hematologic recovery (CRi)** | Achievement of all CR criteria except for hematologic recovery with residual neutropenia (ANC <1 × 10^9^/L) with or without RBC/platelet transfusion independence. |
| **Composite complete remission (CRc)** | Achievement of CR, CRi, or CRp. |
| **Partial remission (PR)** | Presence of regenerating normal hematopoietic cells in bone marrow with evidence of peripheral recovery with no (or only a few regenerating) circulating blasts and with a ≥50% decrease in the number of blasts in the bone marrow aspirate with total marrow blasts between 5% and 25%. A value of ≤5% blasts is also considered a PR if Auer rods are present. |
| **Relapse** | - Relapse after CR, CRh, CRp, or CRi   - Reappearance of leukemic blasts in the peripheral blood and a ≥5% increase in the percentage of blasts that is not attributable to any other cause, or reappearance or new appearance of extramedullary leukemia - Relapse after PR   - Reappearance of significant numbers of peripheral blasts and an increase in the percentage of blasts in the bone marrow aspirate to >25% not attributable to any other cause, or reappearance or new appearance of extramedullary leukemia |
| **Overall survival (OS)** | Time from the date of randomization until the date of death from any cause. For subjects who are not known to have died by the end of study follow-up, OS was censored at the date of last contact. |
| **Event-free survival (EFS)** | Time from the date of randomization until the date of documented relapse (excluding relapse after PR), treatment failure, or death, whichever occurs first. For subjects who are not known to have had a relapse, treatment failure, or death event, EFS was censored at the date of last relapse-free disease assessment. |

**Table S2. Baseline Characteristics of Gilteritinib-Treated R/R *FLT3*^mut+^ AML Patients Without Relapse for 60 Days After HSCT**

| **Characteristic** | **Resumed Gilteritinib**  **(n=35)** | **Did Not Resume Gilteritinib (n=16)** |
| --- | --- | --- |
| **Female, n (%)** | 17 (49) | 6 (38) |
| **Median age, years (range)** | 48 (27-71) | 58 (22-64) |
| **ECOG performance status 0-1, n (%)** | 35 (100) | 16 (100) |
| **Baseline relapsed or refractory status, n (%)** | | |
| Relapsed  Primary refractory | 16 (46)  19 (54) | 9 (56)  7 (44) |
| ***FLT3* mutation type, n (%)** | | |
| *FLT3*-ITD only  *FLT3*-TKD only  *FLT3*-ITD and -TKD | 30 (86)  4 (11)  1 (3) | 12 (75)  2 (13)  2 (13) |
| **Cytogenetic risk status, n (%)** | | |
| Favorable  Intermediate  Unfavorable  Other/Unknown | 1 (3)  28 (80)  1 (3)  5 (14) | 0  12 (75)  1 (6)  3 (19) |
| **Preselected salvage chemotherapy,^a^ n (%)** | | |
| High-intensity chemotherapy  Low-intensity chemotherapy | 30 (86)  5 (14) | 14 (88)  2 (13) |
| **Response to first-line therapy per IRT** | | |
| Relapse ≤6 months after allogeneic HSCT  Relapse >6 months after allogeneic HSCT  Relapse ≤6 months after CRc without HSCT  Relapse >6 months after CRc without HSCT  Primary refractory | 1 (3)  2 (6)  11 (31)  3 (9)  18 (51) | 1 (6)  0  6 (38)  1 (6)  8 (50) |
| **Prior anthracyclines, n (%)** | | |
| Yes  No | 34 (97)  1 (3) | 16 (100)  0 |
| **Prior TKI therapy, n (%)** | | |
| Midostaurin  Sorafenib | 1 (3)  1 (3) | 0  0 |
| **Prior HSCT, n (%)** | | |
| Yes  No | 3 (9)  32 (91) | 1 (6)  15 (94) |
| ***FLT3*-ITD allelic ratio, n (%)** | | |
| High  Low  Missing | 15 (43)  16 (46)  4 (11) | 3 (19)  11 (69)  1 (6) |
| **Co-mutations, n (%)** | | |
| *NPM1*  *DNMT3A*  *NPM1* and *DNMT3A*  *WT1*  *IDH1/IDH2* | 16 (46)  12 (34)  11 (31)  10 (29)  3 (9) | 7 (44)  6 (38)  4 (25)  3 (19)  1 (6) |

^a^Per IRT.

Abbreviations: AML, acute myeloid leukemia; CRc, composite complete remission; ECOG, Eastern Cooperative Oncology Group; *FLT3*, *fms-like tyrosine kinase 3*; HSCT, hematopoietic stem cell transplantation; IRT, interactive response technology; ITD, internal tandem duplication; mut+, mutated; R/R, relapsed or refractory; TKD, tyrosine kinase domain; TKI, tyrosine kinase inhibitor.

**Figure S1. Overall Survival Landmarked to the Date of HSCT Based on Pretransplant Response in Patients With *FLT3*^mut+^ R/R AML**

A. CRc Before HSCT


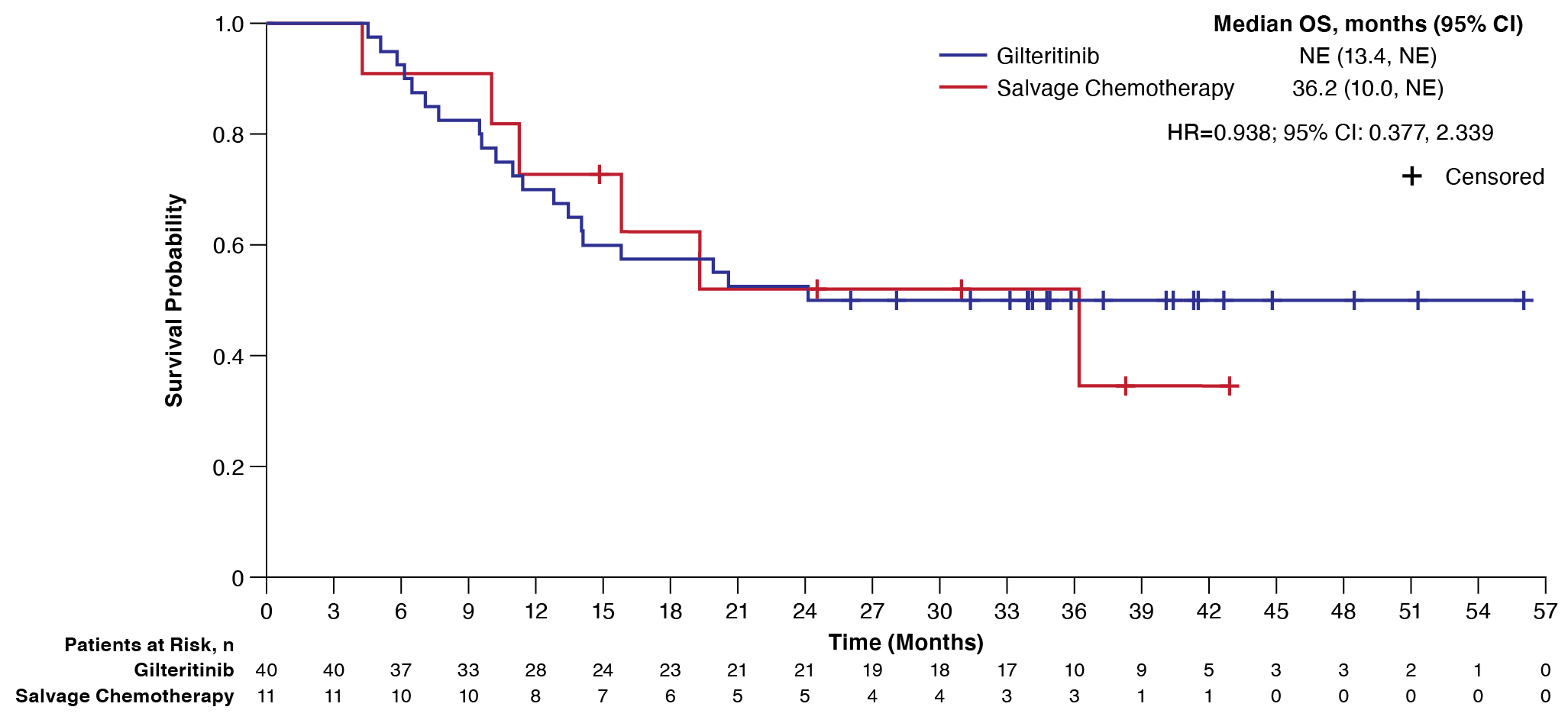


B. Without CRc Before HSCT


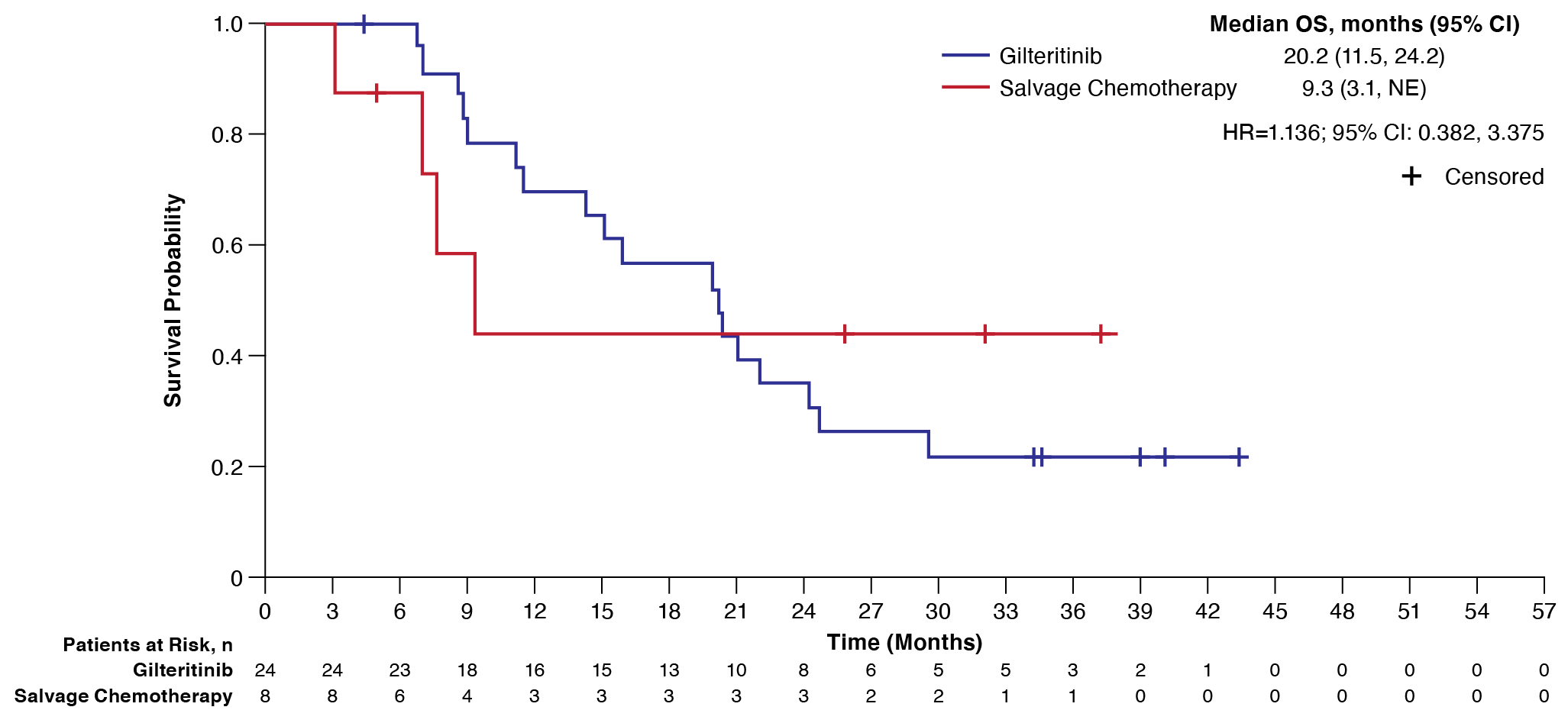


Abbreviations: AML, acute myeloid leukemia; CI, confidence interval; CRc, composite complete remission; *FLT3*, *fms-like tyrosine kinase 3*; HR, hazard ratio; HSCT, hematopoietic stem cell transplantation; mut+, mutated; NE, not evaluable; OS, overall survival; R/R, relapsed or refractory.

**Figure S2. Overall Survival by Remission in Patients Who Achieved CR/CRh Before HSCT Landmarked to the Date of HSCT**


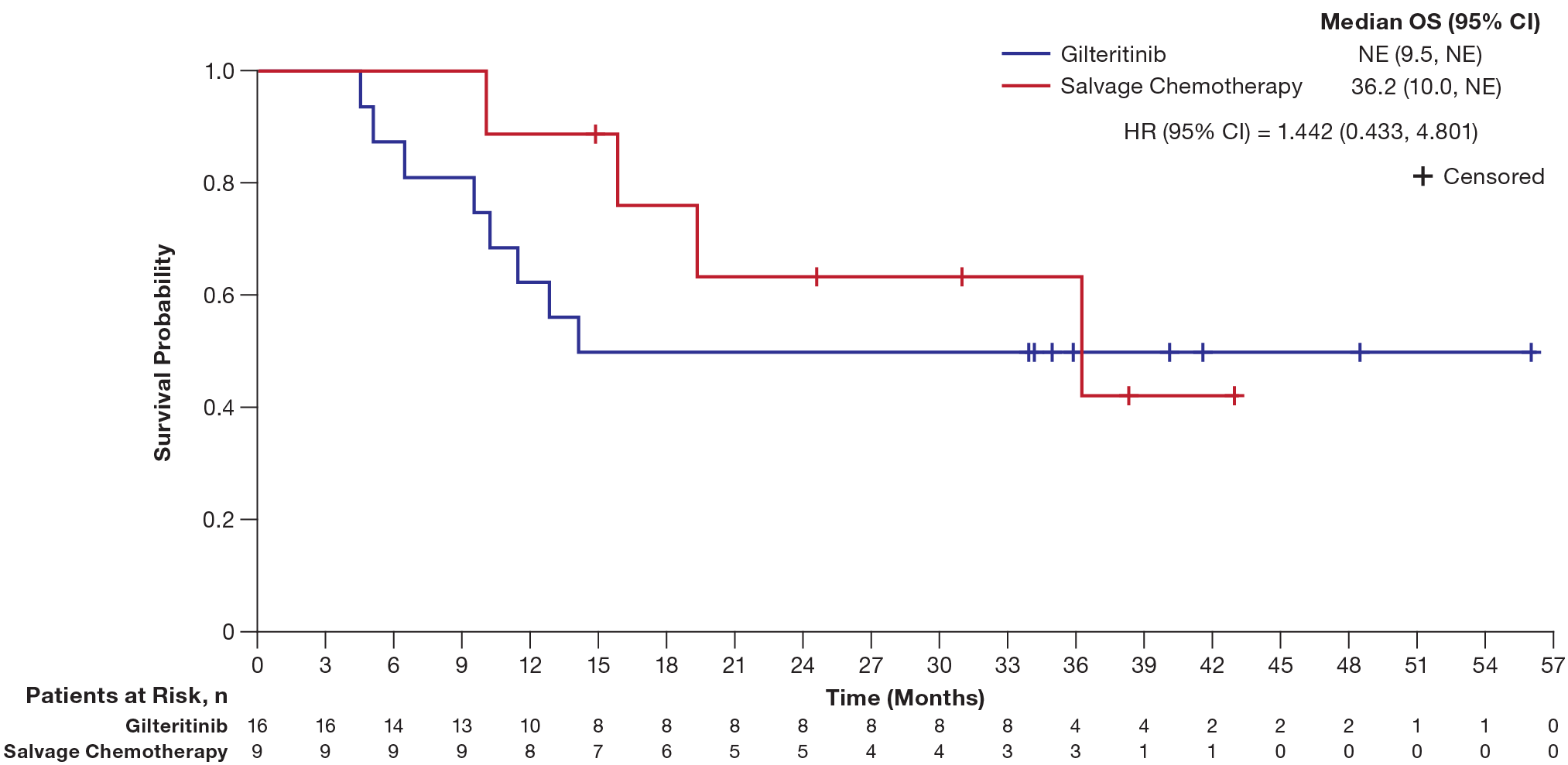


Abbreviations: CI, confidence interval; CR/CRh, complete remission with full or partial hematologic recovery; HR, hazard ratio; HSCT, hematopoietic stem cell transplantation; NE, not evaluable; OS, overall survival.

**Figure S3. Incidence of Posttransplant Grade ≥3 Adverse Events of Interest After Restart of Gilteritinib Therapy^a^**


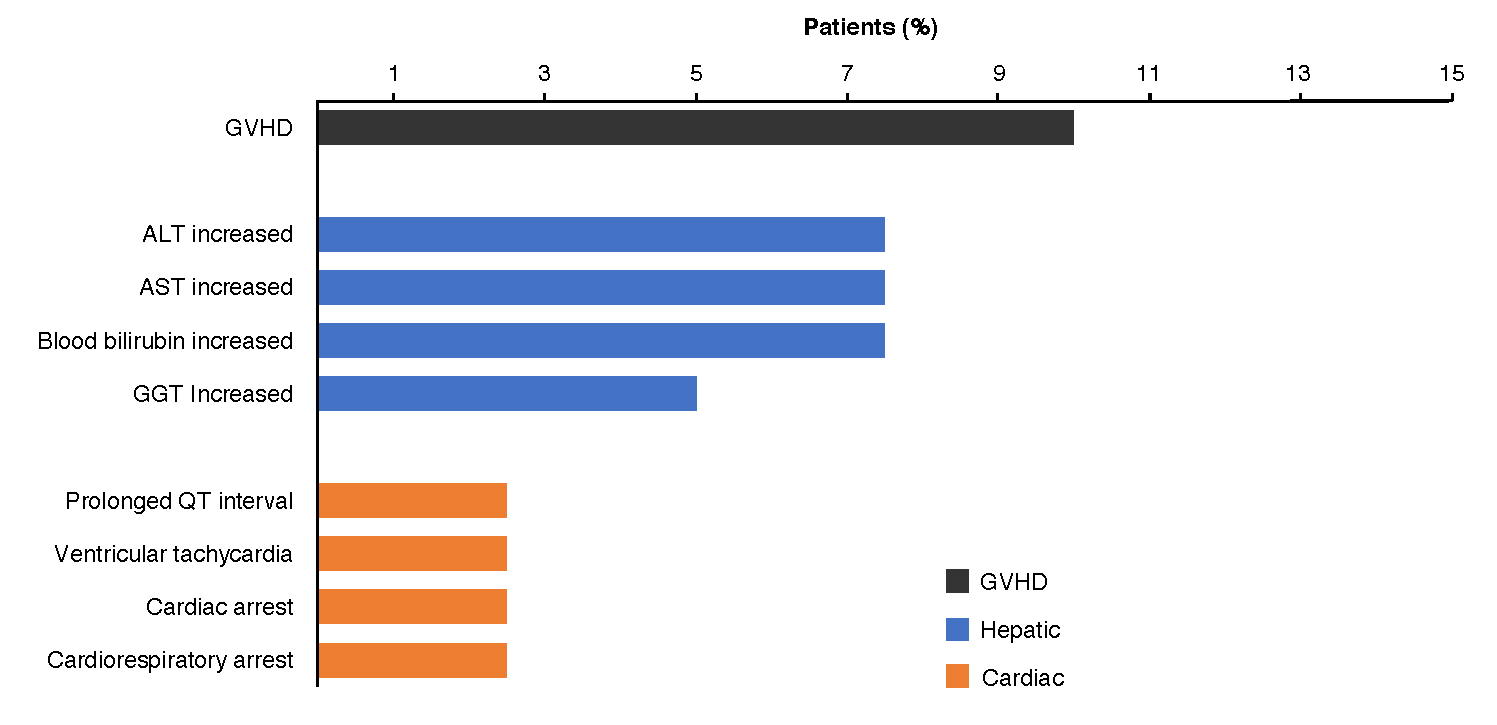


^a^All AEs during study treatment and within 30 days from the last study treatment were reported.

Abbreviations: AEs, adverse events; ALT, alanine aminotransferase; AST, aspartate aminotransferase; GGT, gamma-glutamyl transferase; GVHD, graft-versus-host disease.
